# Species-Specific Differences in the Expression of the *HNF1A*, *HNF1B* and *HNF4A* Genes

**DOI:** 10.1371/journal.pone.0007855

**Published:** 2009-11-16

**Authors:** Lorna W. Harries, James E. Brown, Anna L. Gloyn

**Affiliations:** 1 Institute of Biomedical and Clinical Sciences, Peninsula Medical School, University of Exeter, Exeter, United Kingdom; 2 Aston Research Centre for Healthy Ageing, Aston University, Birmingham, United Kingdom; 3 Oxford Centre for Diabetes Endocrinology and Metabolism, University of Oxford, Oxford, United Kingdom; INSERM, France

## Abstract

**Background:**

The *HNF1A*, *HNF1B* and *HNF4A* genes are part of an autoregulatory network in mammalian pancreas, liver, kidney and gut. The layout of this network appears to be similar in rodents and humans, but inactivation of *HNF1A*, *HNF1B* or *HNF4A* genes in animal models cause divergent phenotypes to those seen in man. We hypothesised that some differences may arise from variation in the expression profile of alternatively processed isoforms between species.

**Methodology/Principal Findings:**

We measured the expression of the major isoforms of the *HNF1A*, *HNF1B* and *HNF4A* genes in human and rodent pancreas, islet, liver and kidney by isoform-specific quantitative real-time PCR and compared their expression by the comparative Ct (ΔΔCt) method. We found major changes in the expression profiles of the HNF genes between humans and rodents. The principal difference lies in the expression of the *HNF1A* gene, which exists as three isoforms in man, but as a single isoform only in rodents. More subtle changes were to the balance of *HNF1B* and *HNF4A* isoforms between species; the repressor isoform *HNF1B(C)* comprised only 6% in human islets compared with 24–26% in rodents (p = 0.006) whereas *HNF4A9* comprised 22% of *HNF4A* expression in human pancreas but only 11% in rodents (p = 0.001).

**Conclusions/Significance:**

The differences we note in the isoform-specific expression of the human and rodent *HNF1A*, *HNF1B* and *HNF4A* genes may impact on the absolute activity of these genes, and therefore on the activity of the pancreatic transcription factor network as a whole. We conclude that alterations to expression of *HNF* isoforms may underlie some of the phenotypic variation caused by mutations in these genes.

## Introduction

The hepatocyte nuclear factors genes, which code for a family of tissue-specific transcription factors, represent one group which exhibit phenotypic divergence between rodent and man. In humans, heterozygous mutations in the genes coding for the HNF1 homeobox A (*HNF1A*), hepatocyte nuclear factor -4 alpha (*HNF4A*) and HNF1 homeobox B (*HNF1B*) genes cause maturity-onset diabetes of the young subtypes *HNF1A*-MODY and *HNF4A*-MODY, and the renal cysts and diabetes (RCAD) syndrome respectively. MODY is characterised by progressive beta-cell failure and an inability to increase insulin secretion in response to hyperglycaemia [1], whereas RCAD is characterised by renal cystic disease, young-onset diabetes, pancreatic atrophy, abnormal liver function tests, and in some cases genital tract abnormalities [2].

Homozygous germline *HNF1A*, *HNF1B* or *HNF4A* mutations have not been found in humans [2], [3]; their associated phenotypes manifest in heterozygous state, whereas mice carrying only one defective copy of the *HNF1A* or *HNF4A* gene show no defects in glycolytic signalling or renal glucose reabsorption as do their human counterparts [4]–[6]. Similarly, the beta cell-specific conditional knockout mouse is only hyperglycaemic during an intraperitoneal glucose tolerance test, whereas RCAD patients have fasting plasma hyperglycemia [7]. These animals also demonstrate no decrease in insulin sensitivity upon glucose challenge when compared to wild-type littermates [8], whereas RCAD patients are insulin resistant [9]. *HNF4A* knockout mice demonstrate altered cholesterol and triglyceride profiles [10], whereas studies of these parameters in human *HNF4A*-MODY patients have been conflicting [11], [12].

Several factors may contribute to the differences in phenotype between the animal models, and human MODY/RCAD patients. Firstly, phenotypic may arise from the timing of gene knockout in the animal models. In the case of the *HNF1B* gene, the conditional deletion was been carried out in adult (or at least partly differentiated) mouse islets, since the insulin gene was used to target the beta cells for gene knockout [8]. The majority of the developmental effects requiring HNF-1β activity would thus have occurred prior to gene knockout, whereas developing islets in RCAD patients would have been exposed to the effects of the mutation from fertilization. Secondly, since MODY is known to be a disorder of haploinsufficiency [13], [14], we cannot rule out the possibility that there may also be species-specific differences in the dosage of the *HNF1A*, *HNF1B* and *HNF4A* gene products that are required for full function.

**Figure 1:**
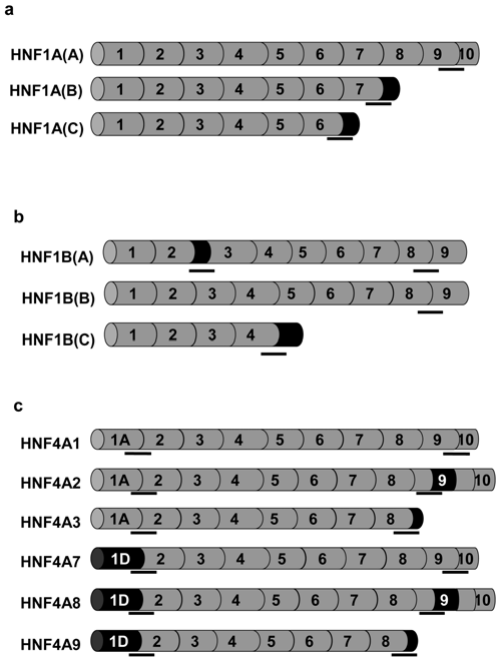
Alternatively processed forms of the *HNF1A*, *HNF1B* and *HNF4A* genes. The structure of alternately processed isoforms of the *HNF1A* (1a), *HNF1B* (1b) and *HNF4A* (1c) genes is given. Exons are indicated in gray and numbered, and isoform-specific novel coding regions in black. The position of the isoform-specific probes are given by black bars.

Variation in expression could be also generated by species-specific differences in the amount and nature of mRNA transcripts produced. Although some alternate mRNA processing events are conserved between species, it has been suggested that a significant number of genes which are known to be alternatively processed in man, do not produce multiple isoforms in rodents [15]. In man, the *HNF1A*, *HNF1B* and *HNF4A* genes produce three, three and nine isoforms respectively by a combination of alternate promoter usage, alternate splicing and differential use of polyadenylation sites; figures 1a–1c [16]–[18]. Although the existence of some of these isoforms have been noted in rodents [19]–[21], to date there has been no systematic comparison of their spatial expression patterns between rodents and man. We therefore aimed to compare the expression patterns of *HNF1A*, *HNF1B* and *HNF4A* isoforms in mouse, rat and human islet, total pancreas, liver and kidney. Differences between species in the site and level of expression may help to explain the phenotypic differences seen in HNF gene knockout animal models and those observed in *HNF1A*-MODY, RCAD and *HNF4A*-MODY patients.

## Results

### Validation of Real-Time PCR for the Detection and Quantification of Alternatively Spliced Transcripts

Real-time PCR assays for human *HNF1B* isoforms and mouse and rat *HNF1A*, *HNF1B* and *HNF4A* isoforms all proved quantitative across a dynamic range of more than seven 1∶2 serial dilutions, with efficiencies of detection ranging from −3.24 to −3.79. The correlations between the crossing point and input template (r^2^ value) were also good with r^2^ values all above 0.90. Human *HNF1A* and *HNF4A* assays have been previously published [16], [17].

### Expression Profile of the Human, Mouse and Rat *HNF1A* Gene

**Figure 2:**
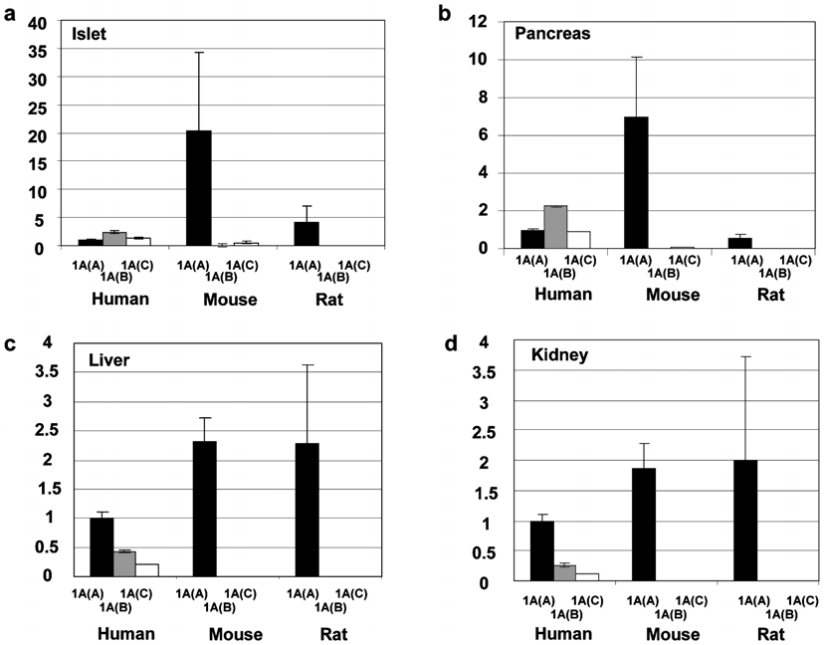
The expression profile of the *HNF1A* gene in human, mouse and rat. The expression profile of the *HNF1A* gene in human, mouse and rat is given. The species is given on the X-axis and the relative quantification of each isoform relative to the B2M gene is given on the Y-axis. Each transcript is normalized to the levels of human *HNF1A(A)* in each tissue. *HNF1A(A)* is given by black bars, *HNF1A(B)* by grey bars and *HNF1A(C)* by white bars. Error bars indicate the upper and lower limits of transcript expression. **a.** islet, **b.** pancreas, **c.** liver, **d.** kidney.

The human *HNF1A* gene produces three isoforms; *HNF1A(A), HNF1A(B)* and *HNF1A[C]*, but in both rodent species the *HNF1A(A)* transcript was essentially the only isoform expressed in islet, pancreas, liver and kidney. The *HNF1A(B)* and *HNF1A(C)* transcripts were present but never comprising more than 3% of the rodent *HNF1A* transcriptome (figure 2a–2d). In all species, *HNF1A* expression was highest in islet and pancreas, and lowest in liver and kidney (figure 2a–2d). Levels of *HNF1A(A)* were markedly higher in rodent tissues than in human tissues.

### Expression Profile of the Human, Mouse and Rat *HNF1B* Gene

**Figure 3:**
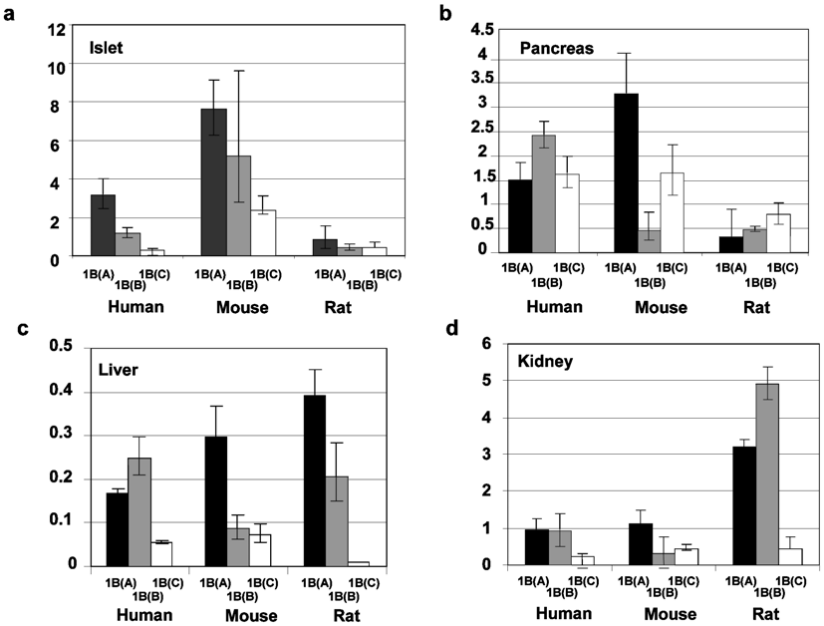
The expression profile of the *HNF1B* gene in human, mouse and rat. The expression profile of the *HNF1B* gene in human, mouse and rat is given. The species and transcript identity identity is given on the X-axis and the relative quantification of each isoform relative to the B2M gene is given on the Y-axis. Each transcript is normalized to the levels of human *HNF1A(A)* in each tissue. Error bars indicate the upper and lower limits of transcript expression. **a.** islet, **b.** pancreas, **c.** liver, **d.** kidney.

Both human and rodent *HNF1B* genes produce three isoforms; *HNF1B(A)*, *HNF1B(B)* and *HNF1B(C*) (figure 3a–3d). Rodent islets expressed less *HNF1B(A)* relative to human islets; comprising 50% and 49% of total *HNF1B* expression in mice and rats respectively, compared to 69% in man (figure 3a; p = 0.004). Rodent islets also had proportionally more *HNF1B(C)* than human islets (16 and 26% for mouse and rat respectively v/s 6% for human; p = 0.006). Overall *HNF1B(B)* was the predominant isoform in human pancreas (figure 3b), whereas in mouse, it was less abundant (44% in man v/s 9 and 31% in mouse and rat; p = 0.003, figure 3b). Although *HNF1B* was expressed only at low levels in liver, the *HNF1B(A)*:*HNF1B(B)* balance is also altered in rodents compared with man (figure 3c). Rodent liver had proportionally more *HNF1B(A)*; comprising 65 and 64% total *HNF1B* expression in mouse and rat liver respectively compared with 36% in man (p = 0.04).

### Expression Profile of the Human, Mouse and Rat *HNF4A* Gene

**Figure 4:**
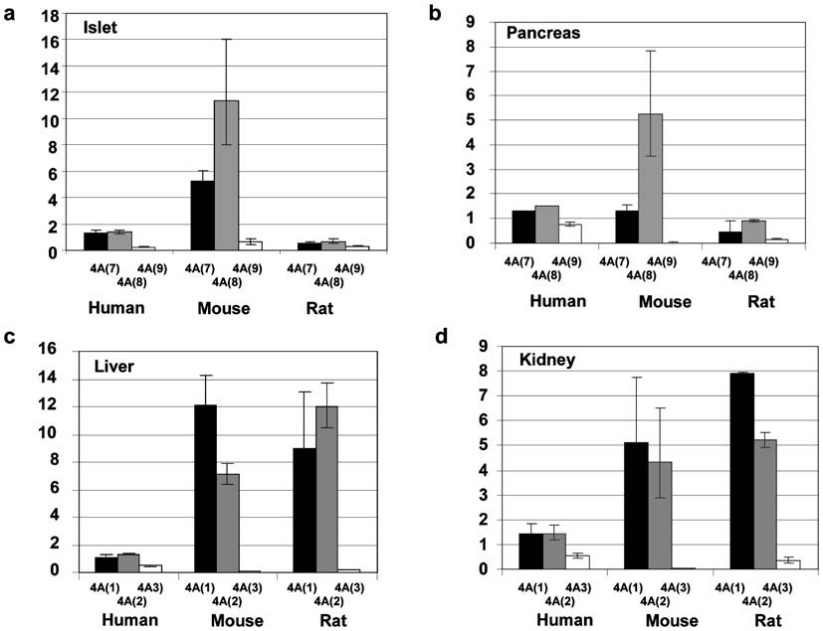
The expression profile of the *HNF4A* gene in human, mouse and rat. The expression profile of the *HNF4A* gene in human, mouse and rat is given. The species and transcript identity is given on the X-axis and the relative quantification of each isoform relative to the B2M gene is given on the Y-axis. Each transcript is normalized to the levels of human *HNF1A(A)* in each tissue. Error bars indicate the upper and lower limits of transcript expression. **a.** islet, **b.** pancreas, **c.** liver, **d.** kidney.

We detected a total of six *HNF4A* isoforms in man, mouse and rat (figure 4a–4d). As noted for *HNF1A* transcripts, levels of *HNF4A* isoforms were markedly higher in rodent tissues than in human tissues. In rodent pancreatic and islet tissue, *HNF4A* expression was driven from the P2 promoter as it is in man (Figure 4a and 4b). In human and rat, *HNF4A7*, and *HNF4A8* transcripts were present in approximately equal quantities in islets, whereas the *HNF4A8* transcript was more prevalent in mouse, comprising 66% of *HNF4A* expression compared with 46% in human islet and 43% in rat; p = 0.003 (figure 4a). *HNF4A9* transcripts comprised a minor transcript in rodent islets, as in human islets [17], although levels were slightly higher in rat comprising 22% of total islet *HNF4A* expression compared with 9% in man and 4% in mouse; p = 0.006. Human pancreatic tissue maintained the equal balance of *HNF4A7* to *HNF4A8* transcripts, comprising 36 and 42% of total *HNF4A* expression respectively (figure 4b), whereas both rat and mouse pancreas demonstrated higher levels of the *HNF4A8* transcript which comprised 80% and 60% of total *HNF4A* expression in mouse and rat respectively compared with 42% in humans (p = 0.002). Human pancreas also contained more *HNF4A9* transcript, relative to rodent pancreas (figure 4b; 22% in the human v/s 0 and 11% in mouse and rat respectively; p = 0.001). Transcripts from the P1 promoter also demonstrated species-specific differences in expression profile. In mouse liver, we found *HNF4A1* transcripts to be more common in mouse than in humans or rats (present at 63% in mice v/s 37% in human and 42% in rat; p = 0.05) (figure 4c). We also noted that *HNF4A3* expression was higher in human liver relative to rodent liver (17% v/s 0% in mouse and 1% total *HNF4A* expression in rat; p = 0.027). This was also evident in human kidney, compared to rodent kidney (figure 4d; *HNF4A3* comprising 16% of total *HNF4A* expression in human, v/s 0% in mouse and 3% in rat; p = 0.039).

### Effect of Dietary Fat and Carbohydrate on HNF Profiles in Rodent Liver

Differences in the HNF expression profile between species could arise from differences in the nature of the nutrient intake in rodent and human subjects. As we were not able to define these precisely, we assessed the possible effect of increased fat and reduced carbohydrate intake on the profile of HNF isoforms in rat liver. We found that dietary manipulation caused no differences in either the level or pattern of *HNF1A*, *HNF1B* or *HNF4A* gene expression.

## Discussion

We have identified significant differences in the expression profiles of the *HNF1A*, *HNF1B* and *HNF4A* genes between human and rodent species. The major difference we identified is in the expression of the rodent and human *HNF1A* genes, which code for three isoforms in humans, but was expressed as a sole isoform, *HNF1A(A)* in all tissues tested in rodents. One potential explanation for the lack of expression of *HNF1A(B)* and *HNF1B(C)* in rodent tissues may lie in sequence divergence from the human sequences in rodents in the area surrounding the polyadenylation signal. The human *HNF1A(B)* and *HNF1A(C)* isoforms utilize the same variant polyadenylation signal (AACAGA) [18], which is not present in the mouse or rat *HNF1A* genomic sequence. Thus, once transcribed, the partially-processed *HNF1A(B)* or *HNF1A(C)* transcripts would lack a poly-A tail, and thus be unstable.

We also detected more subtle differences in *HNF1B* and *HNF4A* profiles, particularly in the relative balance of *HNF4A7* and *HNF4A8* transcripts in the pancreas, and the greater abundance of *HNF4A3* and *HNF4A9* in human tissues. It is possible that that the differences we note could arise from alterations in the profile of *HNF1A*, *HNF1B* or *HNF4A* expression in response nutritional differences between man and rodent, but we found no evidence to suggest that high fat intake or reduced carbohydrate intake influence the HNF profiles.

Our *HNF1B* results also highlight some interesting differences. Previous studies have indicated that *HNF1B* expression is highest in the kidney and lowest in the liver. Our results indicate that both liver and islets contain significant amounts of *HNF1B* mRNA (Figure 3a–d). The reasons for this discrepancy are unclear. There are also indications that the *HNF1B* gene may be under post-transcriptional regulation; previous reports have suggested that although human islets contain significant amounts of *HNF1B* mRNA, levels of HNF-1β in these tissues are barely detectable [22]. This may indicate targeting of *HNF1B* by small regulatory RNA species such as microRNAs.

HNF-1α, HNF-1β and HNF-4α proteins have key roles in embryonic development and in mature homeostasis. These factors exist in a tightly regulated feedback circuit in most tissues with expression, although the precise nature of co-operative regulation may differ between tissues [23]. Therefore, even subtle differences in the relative activity of any of these genes may have profound consequences overall network activity. The relative balance of isoforms may be crucial, since the structural differences between transcripts result in proteins with different properties. Since HNF-1α and HNF1β act as dimers, even small amounts of the variant isoforms could modify total activity *in vivo*. The *HNF1B(A)* and *HNF1B(B)* isoforms are very similar in structure and could therefore demonstrate functional redundancy. Differences in their relative expression levels between species may not therefore have physiological consequences. However, the higher levels of the repressor molecule, *HNF1B(C)* we note in rodent islets compared to human islets, could potentially lead to lower relative HNF-1β activity levels in rodents. The presence of a sole *HNF1A* isoform in rodents may suggest that *HNF1A(B)* and *HNF1A(C)* are not absolutely necessary for function in rats and mice. However, our previous data suggests that *HNF1A(B)*, *HNF1A(C), HNF4A3 and HNF4A9* may have an important role in human beta cell function since their presence can modify MODY phenotype [16], [17].

MODY and RCAD are autosomal dominant disorders, thought to be mediated by a haploinsufficiency-based mechanism due to a reduced amount of HNF-1α, HNF-1β or HNF-4α proteins [13], [14]. The levels of HNF transcription factors present in normal tissue are therefore likely to have an influence on the phenotype produced by inactivity of one or more alleles. Our finding that the overall levels of *HNF1A* and *HNF4A* transcripts were higher in rodent tissues than human tissues may therefore have significance. Since the absolute dosage of the genes in question is crucial, differences to the overall levels of these genes, regardless of isoform profiles, may also have an effect. It may prove to be the case that levels of HNF-1α and HNF-4α are sufficiently high in most mouse and rat tissues that they are above the threshold needed for exhibition of disease phenotype in these animal models.

The differences in *HNF1A*, *HNF1B* and *HNF4A* expression in normal human and rodent tissues has the potential to lead to subtle alterations activity of the transcription factor network. Under normal conditions, human and rodent tissues may not exhibit significant physiological differences. However, species-specific differences in phenotype may become apparent when the system is challenged by alterations to the transcription factor network, either through gene mutation or knockout.

We present here the first direct comparison of the expression patterns of the *HNF1A*, *HNF1B* and *HNF4A* genes in human and rodent species. These findings represent an important difference in mRNA processing of the HNF genes between rodents and man. We therefore hypothesise that some of the phenotypic differences between human MODY and the animal models may arise from alterations to the activity of the transcription factor network between species resulting from differential mRNA processing.

## Methods

### RNA Samples

RNA samples were obtained from kidney (pooled from six Caucasian females, 28 – 52 yrs), liver (pooled from two male Caucasians 51 and 64 yrs), pancreas (pooled from five male and female Caucasians, 24 to 77 yrs) (Clontech, Oxford, UK), and isolated human islets (two male Caucasians 40 and 56 yrs) (NDRI, Philadelphia, USA) and have previously been profiled for the *HNF1A* and *HNF4A* genes [16], [17]. All human samples were from non-diabetic individuals who had died as a result of trauma. Pooled rodent liver, kidney and total pancreas RNAs were from Wistar rats, aged 7–13 weeks or BALB/C mice aged 3–8 weeks old (AMS biotechnologies, Oxon, UK). Pooled mouse islet RNA was derived from NMRI mice aged 12–28 weeks old (n = 4). Pooled rat islet RNA was the gift of Professor Noel Morgan, and was derived from male Wistar rats (n = 6; 180–220 g). All animals had been fed on a standard chow diet. To determine whether dietary intake could have an effect on the expression of HNF isoforms, we also tested RNA from 12–18 week old Wistar rats fed for 10 weeks on either a standard diet (69% carbohydrate, 19% protein, 11% fat, 6.1% sat. fat; n = 3) or on a high fat diet (35% carbohydrate, 20% protein, 45% fat, 17.8% sat. fat; n = 4). RNA samples were DNAse treated prior to reverse transcription using the TURBO DNAse kit (Ambion, Huntingdon, UK).

### Real-Rime PCR Assay Development

Assays to human *HNF1A* and *HNF4A* isoforms were previously published [16], [17]. Assays to the three human *HNF1B* isoforms were designed to the reference sequences (available from http://www.ncbi.nlm.nih.gov). Assays to rodent *HNF1A*, *HNF1B* and *HNF4A* isoforms were designed from sequence alignments of the rat, mouse and human genes according to the Santa Cruz genome browser (http://genome.cse.ucsc.edu/cgi-bin/hgGateway). Primer and probe sequences are given in table 1. Real-time PCR assays were obtained from Applied Biosystems (Foster City, USA). The ubiquitously expressed beta-2 Microglobulin (*β2M*) gene was selected as an endogenous control on the basis that its expression does not differ between the tissues studied, according to publically-available expression data. Assays for this gene (human: Hs00187842_m1, mouse: Mm00437764_m1, rat: Rn00560865_m1) were obtained from Applied Biosystems (Foster City, USA). Assays were validated as previously described [16], [17].

**Table 1:** Table 1. Primer and probe sequences for species-specific assays. Probes are labeled at the 5′ end with 6-fluoresceine (6-FAM) and 3′ with a minor groove binding protein quencher (MGB).

Oligo name	Sequence
Rodent HNF1A(A)F	ACCCTTGCCAGCCTCAC
Rodent HNF1A(A)R	TGGAGGCCTCTGTGTCTGA
Rodent HNF1A(A)P	5′6FAM – CACCAAGCAGGTCTTC – MGB 3′
Mouse HNF1A(B)F	GCCCCTTCATGGCAACCA
Mouse HNF1A(B)R	CTCTCCCAGGCCAACGT
Mouse HNF1A(B)P	5′6FAM – CCCACGGTGAGCATC – MGB 3′
Rat HNF1A(B)F	GAGTCCCTTCATGGCAACCA
Rat HNF1A(B)R	GCTCTCCCAGACCAATGTGAA
Rat HNF1A(B)P	5′6FAM – CCCACGGTGAGCAAT – MGB 3′
Mouse HNF1A(C)F	GACCCACGTTCACGAACAC
Mouse HNF1A(C)R	CACAGGAGCCCCACTCATG
Mouse HNF1A(C)P	5′6FAM – CACCCACTTACCGATAACC – MGB 3′
Rat HNF1A(C)F	CACTAACACGGGTGCCTCTAC
Rat HNF1A(C)R	GCACAGGAGCTCCAGTCA
Rat HNF1A(C)P	5′6FAM – ACCCACTTACCAATGACC – MGB 3′
Human HNF1B(A)F	GTACGTCAGAAAGCAACGAGAGAT
Human HNF1B(A)R	TGACTGCTTTTGTCTGTCATATTTCCA
Human HNF1B(A)P	5′6FAM – CCTCCGACAATTCAAC – MGB 3′
Human HNF1B(B)F	ACGTCAGAAAGCAACGAGAGATC
Human HNF1B(B)R	CCCAGGCCCATGGCT
Human HNF1B(B)P	5′6FAM – TCCGACAGTTCAGTCAACA – MGB 3′
Human HNF1B(C)F	CCAGCTCCTCTCCTCCAAAC
Human HNF1B(C)R	GCAGTGAGGCCCAACCT
Human HNF1B(C)P	5′6FAM – AAGCTGTCAGGTAAGCAA – MGB 3′
Rodent HNF1B(A)F	CGTCAGAAAGCAACGGGAGAT
Rodent HNF1B(A)R	CTGCTTTTGTCTGTCATGTTTCCA
Rodent HNF1B(A)P	5′6FAM – CCTCCGACAGTTCAACC – MGB 3′
Rodent HNF1B(B)F	CGTCAGAAAGCAACGGGAGAT
Rodent HNF1B(B)R	GCGGCGCATCTTCTTGTTG
Rodent HNF1B(B)P	5′6FAM – TCCGACAGTTCAGTCAACA – MGB 3′
Mouse HNF1B(C)F	CCTCACCATCAGCCAAGCT
Mouse HNF1B(C)R	GGTTCCGAGGCAGCTAGAG
Mouse HNF1B(C)P	5′6FAM – TCACTCACCTGACATCTT – MGB 3′
Rat HNF1B(C)F	CTCACCACCAGCCAAGCT
Rat HNF1B(C)F	CCAGTTCTGAGGCAGTGAGA
Rat HNF1B(C)F	5′6FAM – CCTCTACTTACCTGACAACTT – MGB 3′
Rodent HNF4A(1/3)F	CAAGAACACATGGGCACCAATG
Rodent HNF4A(1/3)R	GGTGATGGCTGTGGAGTCT
Rodent HNF4A(1/3)P	5′6FAM – AGCAATGGACAGATGTCCAC – MGB 3′
Rodent HNF4A(2/8)F	GCCCTCTCACCTCAGCAAT
Rodent HNF4A(2/8)F	GGTGATGGCTGTGGAGTCT
Rodent HNF4A(2/8)F	5′6FAM – CCACTCACACATCTGTCC – MGB 3′
Rat HNF4A(3/9)F	GGCATGGCCAAGATTGACAAC
Rat HNF4A(3/9)R	CTACAGCTCCCCAAAGACCAT
Rat HNF4A(3/9)P	5′6FAM – TTGGAGGTCTGTAACTCT – MGB 3′
Mouse HNF4A(3/9)F	CTGCTGCAGGAGATGCTTCT
Mouse HNF4A(3/9)R	CAGCACTACAGATCTCCCAAGAG
Mouse HNF4A(3/9)P	5′6FAM – CATGCATTACAGACCTCC – MGB 3′

### Quantitative Real-Time PCR

Real-time PCR reactions were carried out in triplicate using the ABI Prism 7900HT platform (Applied Biosystems) and contained 5 µl TaqMan Fast Universal Mastermix (no AMPerase) (Applied Biosystems, Foster City, USA), 0.9 µM each primer, 0.25 µM probe and 2 µl cDNA reverse transcribed as above in a total volume of 10 µl. PCR conditions were a single cycle of 95°C for 20 seconds followed by 50 cycles of 95°C for 1 second and 60°C for 20 seconds. The relative expression level of each isoform was then determined relative to the B2M transcript by the comparative (ΔΔCt) method [24]. Transcript abundance was normalized to human *HNF1A(A)* transcript present in each tissue. The expression profiles of the mouse and rat *HNF1A*, *HNF1B* and *HNF4A* genes were then compared with the corresponding profile in the human [16], [17].

### Statistics

All statistical analyses were carried out on pooled samples and measurements thus represent assay replicates rather than biological variation in these samples, which is a limitation of this study. Apparent differences in expression levels between species were tested for statistical significance using a three way comparison of the ΔΔCt values for the test transcript in each species by Kruskal-Wallis test. Pairwise comparisons were achieved using Mann Whitney-U test.
